# Effects of Healthy Ageing on Precision and Binding of Object Location in Visual Short Term Memory

**DOI:** 10.1037/a0038396

**Published:** 2014-12-22

**Authors:** Yoni Pertzov, Maike Heider, Yuying Liang, Masud Husain

**Affiliations:** 1 Department of Psychology, The Hebrew University of Jerusalem; 2 Nuffield Department of Clinical Neurosciences and Department of Experimental Psychology, University of Oxford; 3 Dementia Research Centre, University College London; 4 Nuffield Department of Clinical Neurosciences and Department Experimental Psychology, University of Oxford

**Keywords:** visual short term memory, aging, object-location binding, misbinding, precision, Alzheimer’s disease

## Abstract

Visual short term memory (STM) declines as people get older, but the nature of this deterioration is not well understood. We tested 139 healthy subjects (19–83 years) who were first required to identify a previously seen object and then report its location using a touchscreen. Results demonstrated an age-related decline in both object identification and localization. Deterioration in localization performance was apparent even when only 1 item had to be remembered, worsening disproportionately with increasing memory load. Thus, age-dependent memory degradation cannot be explained simply by a decrease in the number of items that can be held in visual STM but rather by the precision with which they are recalled. More important, there was no evidence for a significant decrease in object-location binding with increasing age. Thus, although precision for object identity and location declines with age, the ability to associate object identity to its location seems to remain unimpaired. As it has been reported that binding deficits in STM might be the first cognitive signs of early Alzheimer’s disease (AD), the finding that object-location binding processes are relatively intact with normal aging supports the possible suitability of using misbinding as an index measures for probing early diagnosis of AD.

Visual short term memory (STM)—the maintenance of visual information in memory over a short period of time while deprived of its direct input—declines during aging (Babcock & Salthouse, 1990; Gazzaley, Cooney, Rissman, & D’Esposito, 2005; Johnson, Logie, & Brockmole, 2010; Jost, Bryck, Vogel, & Mayr, 2011; Reuter-Lorenz & Sylvester, 2005). Age-related decline is of practical importance because STM function plays a crucial role in many cognitive domains such as visual attention (de Fockert, Rees, Frith, & Lavie, 2001; Downing, 2000) and fluid intelligence (Fukuda, Vogel, Mayr, & Awh, 2010). Some studies even claim that fluid intelligence can be improved by practice on tasks that rely on STM processes (Jaeggi, Buschkuehl, Jonides, & Perrig, 2008).

Unfortunately, the underlying cognitive and neural mechanisms responsible for age-related STM decline are not yet clear. The characterization of this deterioration is potentially important, not only for basic understanding of memory and aging but also because it has clinical significance. Studies that have examined how items consisting of several visual features (e.g., color and shape) are remembered have proposed that the process of linking together—or *binding*—the different features is the first to be impaired as a result of Alzheimer’s disease (AD), even when all other standard cognitive tests are within normal range (Parra et al., 2010). More important, it has been claimed that, in contrast, the ability to bind features in visual STM does *not* decline with aging, making such tasks potentially very useful for early screening of AD (Della Sala, Parra, Fabi, Luzzi, & Abrahams, 2012; Parra et al., 2009, 2010).

While some previous studies have shown that binding of visual features remains unimpaired in healthy elderly people (Brockmole, Parra, Della Sala, & Logie, 2008; Parra, Abrahams, Logie, & Della Sala, 2009), a recent Web-based study of more than 55,000 participants reported a significant (but weak) age-related impairment in binding shape and color, in addition to a strong decline in the precision of single-feature memory (Brockmole & Logie, 2013). Another investigation using a dual-feature recall STM task (Peich, Husain, & Bays, 2013) also found a decline in precision for single features as well as within-object binding impairments in elderly people when asked to reproduce from memory both the color and orientation of a colored bar. Therefore, whether an increased level of binding errors is specific to AD or also occurs—at least to some extent—with normal aging is under question.

To better understand age-related changes in STM and distinguish age-related decline from pathologically driven impairments it might be important to investigate a broader range of binding processes in visual memory, such as binding between objects and their locations. Postma (1996) showed that object memory consists of at least two distinct aspects: remembering positions in space and associating which object belongs to each location (what was where). Recent studies have used a novel object-location STM task in which participants are required to report the exact location of previously seen objects using a touch-sensitive screen (see Figure 1). These investigations reported that binding of objects to locations is particularly fragile (Pertzov, Dong, Peich, & Husain, 2012) and specifically impaired in patients with focal damage to their medial temporal lobes, MTLs (Pertzov et al., 2013).[Fig-anchor fig1]

Here we used this task to examine how object-location binding performance is influenced by aging. If object-location binding performance deteriorates with age, it would mean that aging seems to affect a wide range of binding processes in visual STM (Brockmole & Logie, 2013; Peich et al., 2013). On the contrary, if age does not affect object-location binding, this would suggest the potential suitability of this kind of binding test for diagnosis of memory disorders in neurodegenerative diseases. So far, reports regarding age-related deficits in *object-location* binding have been mixed. Olson and colleagues (2004) did not find age-dependent difficulties in a test of configural binding (how different locations are bound to each other) after short delays of ∼1.5 s. These findings suggest that initial encoding of spatial information for relatively small numbers of items is largely preserved in healthy older adults.

However, another study that used slightly longer retention intervals did find a decrement in performance (Mitchell, Johnson, Raye, Mather, & D’Esposito, 2000). Mitchell and colleagues (2000) presented three line drawings successively, each in a different cell of a 3 × 3 grid, and participants had to maintain the information over an 8-s unfilled delay interval. They were tested for which locations were filled, which objects were seen, or which object was displayed at which location. Older adults did not differ significantly from young adults on either item or location information, but were impaired on combination trials. Note, however, that the combined condition was also more difficult than the location condition and was tested in different blocks. A possible way to reconcile the above conflicting reports is if the age-related deficits in binding to locations are not in the encoding stage (and therefore, not affected in short delays) but rather in retaining information over time and, therefore, revealed in longer delays (although see Noack, Lövdén, & Lindenberger, 2012). Indeed, a recent study suggested that extended delay degrades object localization performance in elderly subjects much more than in younger ones (Oosterman et al., 2011).

Therefore, we decided to use various maintenance intervals to investigate whether different delays impact on the rate of forgetting over the life span. Furthermore, in contrast to most previous studies on location memory, we used an analog rather than a discrete measure of report that has been shown to be sensitive to the quality of location memory as well as to object-location binding in the same set of trials (Pertzov, Dong, Peich, & Husain, 2012; Pertzov et al., 2013). This allows us to probe the *quality* of memory (Ma, Husain, & Bays, 2014), rather than the *number of items* remembered, which is the traditional index. More important, it allows us to examine whether recall of even one item is affected by age.

In summary, the study goal was to reach a better understanding of the deterioration in object-location STM as a result of aging, focused on the effects of delay (forgetting) and the *quality* of memory, indexed by precision of recall. Characterizing the baseline patterns of change because of healthy aging would assist in understanding the cognitive impairments in neurodegenerative diseases.

## Material and Methods

### Participants

In total, 139 people (77 females) within an age range 19–83 participated. They were either registered in the subject pool of Psychology departments at UCL and Oxford or responded to printed advertisements in elderly community centers in Oxford and London. All participants reported normal or corrected-to-normal visual acuity and no color blindness. All subjects provided written informed consent before participation. The study was approved by the local ethics committees.

Table 1 provides a short summary of demographics and neuropsychology results (Mini-Mental State Examination and digit span: forward and backward). For the purpose of the analysis we arranged all subjects according to their age and binned them into four equal-sized groups. Initial analysis was performed on these age groups using an omnibus analysis of variance (ANOVA) to guide a more in depth investigation of age effects by correlating individuals’ performance to their age across all participants.[Table-anchor tbl1]

### Stimuli and Procedure

**Figure 1 fig1:**
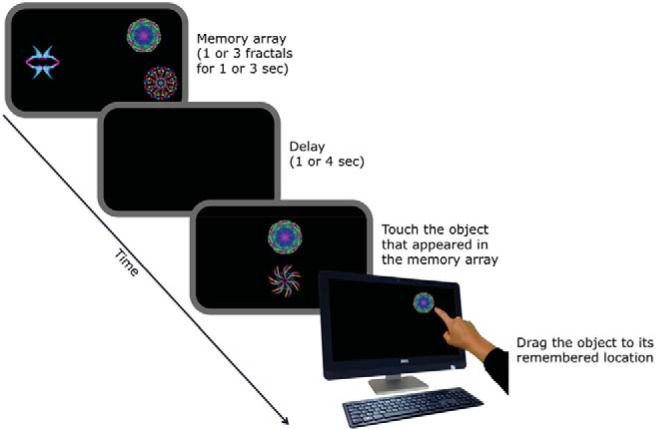
Procedure of a three-item trial: After the initial presentation of three items and after a delay (1 or 4 s) subjects had to recognize and touch the target that had appeared in the memory array of this trial and then drag it to its original location from the memory array. See the online article for the color version of this figure.

We used a recently introduced paradigm (Pertzov et al., 2012, 2013) assessing participants’ ability to remember both the identity and location of objects. Figure 1 shows a scheme of the paradigm.

Participants started each trial manually by pressing space bar. They were then presented with 1 or 3 fractal objects for 1 or 3 s, respectively (to allow thorough encoding), and were asked to remember both identity and location of all objects. This was followed by a delay of either 1 or 4 s after which 2 fractals appeared on the vertical meridian of the screen. One of them had been presented in the initial memory array (target) and the second one was a lure (foil) that had not. Participants had to recognize the target and touch it on the screen. After this they had to drag it to the position on the screen where it had originally been presented in the memory array. Thus, memory for the identity of the objects and their locations could be extracted separately from the two alternative forced choice (identification) and move to remembered location (localization) stages, respectively. Once the participant was satisfied with the chosen location she clicked the space bar button of the keyboard and the next trial was initiated. Localization performance was only included in the analysis if the correct object was identified.

Stimuli were selected from 60 fractals (http://sprott.physics.wisc.edu/fractals.htm) that were always presented on a black background. They were randomly selected but without repetitions within a trial. Each fractal was presented 2 to 3 times per block. The fractals had a maximal width and height of 120 pixels (corresponding to 4° visual angle). In trials where three items were presented every fractal had the same likelihood to be tested. The fractal’s location was determined by a Matlab script (MathWorks) in a pseudorandom manner with several restrictions: All objects had a minimum distance of 9° visual angle from each other to avoid spatial crowding and enable further clear-cut analysis of the localization performance. Furthermore, each fractal had a minimum distance of 3.9° from the edges of the screen and a minimum distance of 6.5° from the center of the screen.

Participants were allowed to sit at a convenient distance from the screen (∼42 cm) to enable comfortable report via the touch screen (Inspiron All-in-One 2320; DELL) with a 1,920 × 1,080 pixel matrix (corresponding to 62° × 35° visual angle). They performed two blocks of 50 trials each. The effect of practice between blocks is reported and discussed in the online Supplemental materials. We decided not to include it in the main text to keep the text fluent and clear. A block included 10 trials with one item to-be-remembered and 40 trials with three items to-be-remembered. The delay was 1 s in 50% of trials and 4 s in the rest. Every subject performed a practice block of 10 trials before starting the experiment ensuring that they had understood the task completely and seen every condition at least once.

Localization memory was computed by taking the distance between the center of the location chosen by the participant and the center of the target location in the initial memory array. For reasons discussed later we also calculated the distance between the location subjects had chosen for the target and the locations of nontargets (unprobed fractals) from the initial memory array. For convenience, and in accordance with visual neuroscience conventions, we converted all localization error values into degree of visual angle for a viewing distance of 42 cm.

To quantify *misbinding errors* we calculated the rate of “*swap errors*” for each participant. A swap error occurred when the target was identified correctly but subsequently placed within a 4.5° radius of one of the nontarget locations. We chose this limit because two fractals were never located closer than 9° of visual angle from each other. This ensured that a fractal could be considered as swapped to only one location at a time and also allows easy comparison with previous data sets using the same task in different populations (Pertzov, Dong, Peich, & Husain, 2012; Pertzov et al., 2013). Analysis of the data using a stricter decision boundary of 4° left the pattern of results unchanged.

For the purpose of the analysis we arranged all subjects according to their age and binned them into four equal-sized groups. Initial analysis was performed on these age groups using an omnibus ANOVA with factors of age-group, delay, and number of items. We used this analysis to guide a more in depth investigation of age effects by correlating individuals’ performance to their age across all participants.

### Neuropsychology

In addition to the main experiment participants performed some established neuropsychological tests. We applied the Mini Mental Status Examination (Folstein, Folstein, & McHugh, 1975) as well as the forward and backward digit span tasks (*N* = 90). In the digit span tasks, participants heard sequences of digits (e.g., 4–2–7–3–1) and had to repeat them verbally either in the same order (forward digit span) or the reverse order (backward digit span). The test started with two sequences of two digits and after successful completion of at least one of the two sequences, two new sequences with one more digit were presented. Participants continued until they reported both sequences of a certain length incorrectly. We scored 1 point for a correctly recalled digit sequence. There were no significant differences between the age groups on either educational level (*F*(3, 111) = 2.0, *p* = .11; one-way ANOVA), MMSE (*F*(3, 84) = 0.5, *p* = .66), forward (*F*(3, 85) = 1.1, *p* = .36), or backward (*F*(3, 85) = 0.9, *p* = .44) digit span performance.

## Results

### Object Identification

**Figure 2 fig2:**
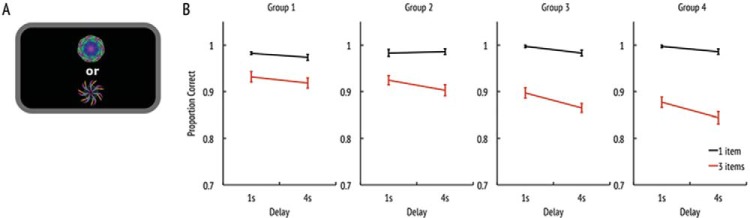
Identification accuracy. (a) Schematic representation of the two-alternative forced-choice identification task. (b) Proportion of trials where the target was identified correctly shown for each of the four different age groups. The *x*-axis represents the delay duration. Black lines denote one item conditions; red (gray) ones indicate three item conditions. See the online article for the color version of this figure.

Object identification performance (see Figure 2) was computed by dividing the number of trials in which the target was identified correctly by the total number of trials. The mixed-design ANOVA showed an overall effect of age-group (*F*(3, 135) = 4.0, *p* < .01, ç_*p*_^2^ = 0.08) with younger adults performing better than older adults. Identification performance was worse when the delay time was long than when it was short (*F*(1, 135) = 21.4, *p* < .001, η_*p*_^2^ = 0.14) and decreased when three items had to be remembered compared with only one item (*F*(1, 135) = 331.1, *p* < .001, η_*p*_^2^ = 0.71).[Fig-anchor fig2]

Furthermore, more items had a stronger deteriorative effect on older people as reflected by a significant interaction between age-group and number of items (*F*(3, 135) = 12.5, *p* < .001, η_*p*_^2^ = 0.22). Finally, a longer maintenance delay had a stronger detrimental effect on more items to-be-remembered than if only one item has to be memorized (Delay × Items: *F*(1, 135) = 6.1, *p* < .05, η_*p*_^2^ = 0.04). Note, however, that identification performance was close to perfect for the one item condition. Therefore, the Delay × Items and Age × Items interactions could be explained by a ceiling effect in the one item condition. No important theoretical conclusion rides on these interactions. All other factors did not reach significance. To conclude, identification performance worsened with longer delays and more items in memory. Older adults were worse on the identification task than younger ones and this pattern was especially pronounced when three items had to be remembered, but did not interact with retention interval.

### Localization Performance

#### Absolute localization error

**Figure 3 fig3:**
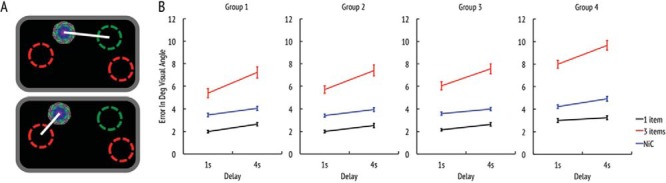
Localization performance. (a) Schematic representations of the different measures (upper figure: error in a three-items trial; below: NIC to the closest object. Green (dark gray circles filled with white) circle represent the target’s original location and red (bright gray) circles the nontarget locations. (b) Localization error for the four different age groups: The *x*-axis represents the maintenance delay. Red (gray) are three items conditions; black are one item conditions; blue (dashed) lines are nearest item control (NIC) measures. See the online article for the color version of this figure.

Absolute localization error (see Figure 3) was measured as the difference between the reported location of a fractal and its true location in the memory array. Error was significantly larger in older than in young participants (*F*(3, 135) = 10.8, *p* < .001, η_*p*_^2^ = 0.19), increased when the delay interval was longer (*F*(1, 135) = 159.6, *p* < .001, η_*p*_^2^ = 0.54), and when more items had to be remembered (*F*(1, 135) = 767.3, *p* < .001, η_*p*_^2^ = 0.85).[Fig-anchor fig3]

The ANOVA revealed a significant interaction between age-group and number of items (*F*(3, 135) = 5.1, *p* < .01, η_*p*_^2^ = 0.10) revealing that the detrimental effect of more items in the memory array was significantly more pronounced in older than in young participants. Replicating previous studies (Pertzov et al., 2012), the increase in localization error with longer maintenance intervals was even more accentuated when three items had to be remembered compared with one item, as reflected by a significant Delay × Items interaction (*F*(1, 135) = 51.2, *p* < .001, η_*p*_^2^ = 0.28).

#### Localization error controlling for swap errors

Next we wanted to know how precisely people remember locations *regardless of the identity* of items. For this, we computed a measure of precision taking into account the fact that observers might sometimes identify the correct fractal but relocate it to a position of one of the other (nonprobed) items in memory, or a nontarget. We classify these mistakes as swap errors. Such swap errors would elicit big error values for absolute localization performance (= distance to target location) despite the fact that location of the chosen nontarget position might be remembered with high precision. From this perspective, the actual localization error reflects not a lack of precision for the target location, but a decision to report the location of a nontarget item. Therefore, we computed the distance between the chosen location and the location of the *closest fractal* in the original memory array, regardless of whether or not it was the target (see Figure 3). We term this measure “*nearest item control*” (NIC).

Contrary to absolute localization performance, there was no significant Age-group × Number of items interaction for NIC (*F*(3, 135) = 0.1, *p* = .984, η_*p*_^2^ = 0.001). This finding suggests that when the identity of the items is rendered irrelevant for purposes of analysis, the amplified deteriorative impact that more items have on older people’s memory disappears. Similar to raw localization performance, the NIC analysis revealed significant effects of delay (*F*(1, 135) = 161.0, *p* < .001, η_*p*_^2^ = 0.54), number of items (*F*(1, 135) = 595.6, *p* < .001, η_*p*_^2^ = 0.82), and age-group (*F*(3, 135) = 9.5, *p* < .001, η_*p*_^2^ = 0.17).

#### Correlation analyses across age

**Figure 4 fig4:**
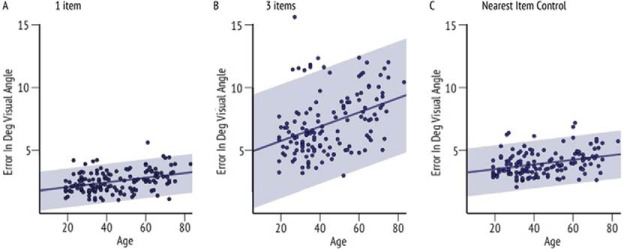
Precision of recall with increasing age. (a) Linear regression between age and localization error when one item has to be remembered, (b) when three items have to be remembered, and (c) for nearest item control (NIC) values. There is an age-related increase of localization error when only one item and three items are remembered, as well as an increase of NIC values (localization error controlled for object-location swapping). The 95% confidence intervals are marked in gray shade. See the online article for the color version of this figure.

To further investigate memory performance without the arbitrary division into four age groups, we performed several subject-wise correlation analyses. First, linear regression of age and localization performance (Figure 4; combined across delays) revealed an increase in localization error in older people even when only one item had to be remembered (*r* = .359, *p* < .001).[Fig-anchor fig4]

Furthermore, as expected, there was also a stronger increase in localization error with increasing age when three items had to be remembered (*r* = .383, *p* < .001). NIC error also increased with age (*r* = .312, *p* < .001). Together these analyses suggest there is a decrease in the precision with which location is recalled as people get older, regardless of object identity memory.

**Figure 5 fig5:**
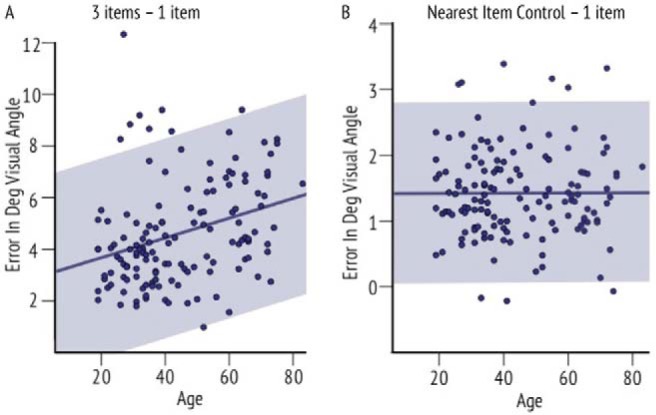
(a) Linear regression between age and difference in localization error between three items and one item to-be-remembered. (b) Linear regression between age and difference in localization error between NIC and one item to-be-remembered. The 95% confidence intervals are marked in gray shade. See the online article for the color version of this figure.

To further investigate the significant interaction of Age-group × Number of items evident in absolute localization performance but not in NIC (where we controlled for swap errors: the possibility of misreporting a nontarget location while correctly identifying the target identity) we attempted to quantify the additional localization error that was caused by more than one item in memory. First the *difference in error* between the three items and one item conditions for absolute localization error was computed for every participant and correlated with age (Figure 5a). Next, we calculated the *difference in error* for NIC (in three item condition—the only condition it could be computed) and one item absolute error, and correlated this with age (Figure 5b).[Fig-anchor fig5]

Although there was a significant positive correlation between age and the difference between three and one item conditions for localization performance (*r* = .310, *p* < .001), the relationship was close to zero for age and difference between NIC and the one item condition (*r* = .001, *p* = .493). The above results were replicated using a partial correlation analysis. Correlation between age and performance on three item condition was significant even when controlling for one item performance (*r* = .22, *p* = .01). On the other hand, correlation between age and performance on NIC was insignificant when controlling for one item condition (*r* = .07, *p* = .38). These results might suggest that the underlying reason for increased localization error in older adults when three items have to be remembered is related to memory being systematically corrupted by misreporting positions of nontarget items. This could be a result of a deficit in object-location binding. However, such an interpretation assumes that participants correctly remembered the identity and location of objects but failed to remember the correct links—*bindings*—between them.

Even though we only used trials in which targets were correctly identified it is possible that in some trials participants only guessed correctly which item was displayed earlier. In such cases, assuming that they still remembered the locations of all fractals, they would still often report the wrong locations simply by chance. To control for trials in which the correct object was selected simply by chance, we performed partial correlation controlling for the *number of identification errors* each participant made. This analysis now yielded close to zero correlations for both three items minus one item (*r* = .074, *p* = .193) and NIC minus one item (*r* = −0.093, *p* = .138).

Thus, although the increased localization error in the elderly when more than one item had to be remembered might indeed be related to increased swap errors, such errors might arise from failures to remember the identity of the items in memory rather than from a true binding deficit. To further investigate this we next directly analyzed swap error performance.

### Swap Errors

We attempted to explicitly quantify participants’ swap error rates by counting the trials where the target was correctly identified but subsequently localized within a radius of 4.5° visual angle of one of the nontarget positions. Recall that each item in the memory array was separated from another one by a minimum of 9° so 4.5° provides a conservative window to classify swap errors. Analysis of the data using a stricter decision boundary of 4° left the pattern of results unchanged.

**Figure 6 fig6:**
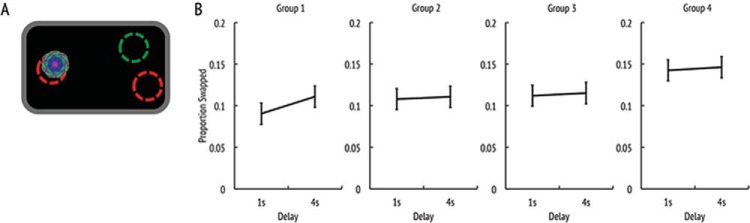
Swap errors for different age groups. (a) Schematic representation of a swap error (green [dark gray filled with white] circle represents the target’s original location and red [light gray] circles the nontarget locations). (b) Proportion of trials where the target has been located to a nontarget location for the four different age groups. The *x*-axis represents the maintenance delay. See the online article for the color version of this figure.

First, we examined performance across our four age groups. The analysis revealed a significant effect of age-group (*F*(3, 135) = 3.4, *p* < .05, η_*p*_^2^ = 0.07) such that people made more swap errors with age (see Figure 6).[Fig-anchor fig6]

**Figure 7 fig7:**
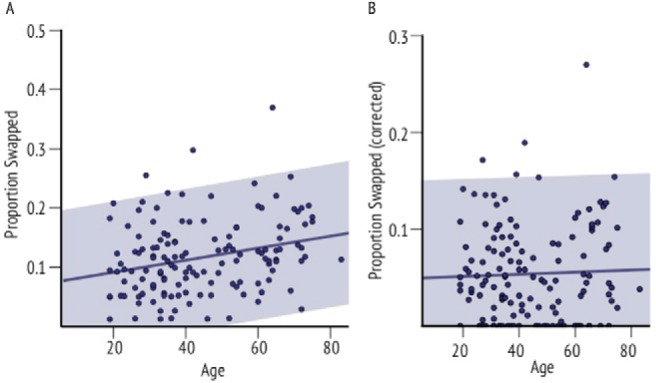
Swap errors and aging. (a) Linear regression between age and overall proportion of trials where swap errors occurred. (b) Linear regression between age and corrected swap error rate. The 95% confidence intervals are marked in gray shade. See the online article for the color version of this figure.

To further investigate age effects on the swap error rate we first collapsed the swap errors across the different delay times and then computed correlations between swap rates and age (Figure 7a). This yielded a significant correlation between the proportion of trials in which swap errors appeared and age (*r* = .265, *p* < .01), suggesting that elderly people did indeed produce more swap errors than younger adults.[Fig-anchor fig7]

However, as mentioned previously, swap errors might occur because participants did not remember the target’s identity and chose the correct target by chance. In such trials, assuming intact location memory participants are expected to place the fractal around a nontarget location in two-thirds of those trials (in one-third of the trials they would localize the fractal near its original location, by chance). Using this assumption, we calculated the upper limit of swap errors that could be explained by failure to remember the identity of the fractal. First, we multiplied identification error rate (this corresponds to highest assumed correct guessing rate) of each subject in each condition by two-thirds (likelihood that one of the two nontarget positions was chosen). Then we subtracted this value from the swap error rate. ANOVA on this index that is a *corrected swap error rate* (controlling for chance guessing of correct identity) no longer yielded a significant effect of age-group (*F*(3, 135) = 0.7, *p* = .568, η_*p*_^2^ = 0.02).

Consistent with the disappearance of the age-effect in the ANOVA, the subject-wise correlation between age and *corrected swap error rate* was not significantly different from zero (Figure 7b; *r* = .037, *p* = .332). Finally, we also performed a partial correlation between age and swap error rate controlling for identification errors. This correlation was small and not significantly different than zero (*r* = .121, *p* = .079). Thus, overall the results suggest that although there is an increase in the proportion of swap errors with increasing age, this could be explained simply by higher identification error rate in older adults. Crucially, when this is carefully controlled, swap errors—or misbinding object identity and location—do not increase with age.

## Discussion

In this study we examined the effects of aging on people’s ability to recall which objects were previously displayed where. There was a decrease in precision of reports for both object identity and location with age. Older adults also had higher probability to swap the location of objects and hence to report the locations of the wrong items in memory. However, this tendency could be explained by forgetting the identity of objects rather than by a failure to bind object identity to location.

It is important to keep in mind that localization precision is an analogues measure and, therefore, much more sensitive than the discrete measure of swap error. This difference can potentially explain why we found age related degradation in localization performance, but not in the swap errors (when controlling identification errors). The age-related differences in object identity shown here may be related to older adults’ tendency to be supported (at least implicitly) by contextual reinstatement. Such support was lacking here because the probe object at the test phase always appeared in the center and not at the position in which it appeared in the study phase.

The duration of the maintenance interval was an important determinant of identification performance, localization performance, and swap errors. Age-related decline was evident also in the 1 s delay and did not deteriorate much further in the next 3 s. This suggests that the age-related impairment might be in encoding to or retrieving from memory, rather than in maintaining information over time. Nevertheless, it is possible that longer delays than those used in our study might have differential effects on different age groups.

In apparent contrast to our findings, Mitchell, Johnson, Raye, Mather, and D’Esposito (2000) reported an increase in misbinding with increasing age and no age-related changes in the precision with which object identity and position are remembered. Several differences between experimental designs might have led to this discrepancy. The first concerns the sensitivity of localization measures: we used a continuous, analog response while in Mitchell et al. (2000) were allowed only two responses (same or different). The increased level of information per trial might explain why we detected changes in localization precision whereas they did not. Second, the maintenance intervals, we used delays of 1 and 4 s whereas Mitchell et al. (2000) used a delay of 8 s. It is possible that the age-dependent binding deficits they reported emerges at longer delays because of maintenance deficits. Third, we used complex, hard-to-name, unfamiliar fractal objects whereas Mitchell and colleagues used simple familiar objects. It is possible that younger participants can utilize verbal strategies to help in remembering object-location associations. However, when objects are not easily linked to verbal tags, younger people might not benefit any more from such an advantage and have to rely on visual memory similarly to elderly participants.

Finally, there is the issue of the exact measure used to capture binding errors. Mitchell et al. (2000) used a paradigm where subjects had to detect changes between subsequently presented memory and test arrays. In the conjunction condition that was used for probing binding errors, features swapped between different objects, and participants had to detect those changes. It has been hypothesized that older people might rely more on familiarity (“I have seen this stimulus before”) rather than recall the exact feature combinations when responding to such arrays (Cowan et al., 2006; Old et al., 2008). This would increase false alarm rates in elderly people because they might make decisions based on the presence or absence of features and report swapped features (binding condition) as no-change, but accurately report introduction of new features (feature condition).

In contrast, in our paradigm participants first had to identify the target and then reproduce its original location on the screen. Thus, young and older adults were not likely to rely on different familiarity-based strategies because they were forced to recall the location of the target object. This enabled us to define swap errors as the misplacement of the target within a predefined radius around one of the nontargets. Thus, our misbinding measure controls for both young and older participants applying familiarity rather than recollection strategies and might therefore, be more direct and sensitive than ones used previously.

Parra et al. (2009, 2010) have suggested that object-location binding might be more affected by age than surface feature binding (color-shape or color-color) as it relies more on the hippocampus than on parahippocampal regions. Because the hippocampus exhibits stronger age-related degeneration than parahippocampal regions (Insausti et al., 1998; Mitchell, Johnson, Raye, & D’Esposito, 2000), aging was speculated to affect primarily object-location binding but not color-shape binding (Insausti et al., 1998; Mayes, Montaldi, & Migo, 2007). In our experiment the age-dependent increase in swap errors disappeared when the deficit in remembering the identity of the fractals was controlled. Thus, although normal ageing leads to some degree of hippocampal atrophy (Grady, 2008), age-related decline in our study was mainly reflected by impaired memory for object identity, importantly without any additional association between object-location binding and aging. A similar view also emerges from recent studies of object-feature binding in STM. An initial study reported that aging is not associated with feature-feature binding, when taking into account a strong age-related decline in feature memory (Brockmole et al., 2008). A subsequent investigation of 55,753 participants found that feature-feature binding errors explain less than a fifth of the age-related variance compared with feature memory (Brockmole & Logie, 2013).

Several other studies on binding in memory (e.g., Brockmole & Logie, 2013; Brockmole et al., 2008; Parra et al., 2009) have used a different strategy to the one used here. By comparing two conditions—one in which features had to be bound to one in which they did not—they provide one type of metric of misbinding errors. Our findings using a single condition to extract different types of error—identification, localization and swap errors. The method we use is complementary to the dual condition tasks and has previously been used to show that swap errors increase over maintenance delays in healthy people, so cannot solely be attributed to deficits at encoding (Pertzov et al., 2012). It is indeed interesting that studies using both types of task come to similar conclusions: no significant increase in misbinding with healthy ageing, strengthening the original claim on this issue (Brockmole & Logie, 2013; Brockmole et al., 2008; Parra et al., 2009).

Of interest to the authors, and in agreement with recent findings by Peich et al. (2013), the analog measurement of the localization performance used here showed that elderly adults exhibit decreased precision in reporting a specific property of a previously seen item (in our case, location) even when only one item has to be remembered. Peich et al. reported similar results for recalling color and orientation of colored bars. Taken together, the findings from both studies suggest that the general impairment older people show occurs not only when large numbers of objects or features have to be remembered, but also when a relatively small amount of information has to be maintained. Thus, age-dependent memory degradation is not explained simply by a decrease in the number of items held in visual STM, but rather in the precision with which they are remembered. Nevertheless, in the study reported here, additional memory load had an even more detrimental effect on older than young people’s performance in both identification and localization parameters. Thus, there might be additional capacity impairment in old people reducing their performance disproportionately as memory load increases. This age-related detrimental effect might also be related to other age-related factors, such as decreased discriminability of locations. Peich et al. also reported that there is deterioration in the ability to bind color and orientation with aging. In the current experiment that used a very different paradigm, after correction for losses in identity memory, we did not find direct evidence for age-related deterioration in objet-location binding.

The lack of an age effect in digit span performance suggests that our group of subjects might consist of an atypical sample of old adults. The older adults that volunteered might perhaps be “overmotivated” or with above normal capabilities. However, lack of age effect in digit span tests is not without precedence (Villardita, Cultrera, Cupone, & Mejìa, 1985) and the digit span results were not strongly correlated with our memory measure (mostly below 0.1, all below 0.3, on Pearson’s correlations). Therefore, it seems unlikely to be a major confound that hampers our conclusions. The fact that some of our measures are strongly influenced by age, unlike the digit span measures, emphasizes the sensitivity of the touchscreen task relative to conventional memory tests.

Characterization of the patterns of change in visual STM with age can potentially be of clinical importance. Parra et al. (2010) reported an impairment in color-shape binding performance in patients with familial AD, but also in asymptomatic mutation carriers (aMC) who had not yet developed any sign of AD or mild cognitive impairment. They have proposed that deficits in binding might be the first subtle signs of cognitive decline in AD and might be used for early diagnosis or for monitoring response to treatments (Parra et al., 2009, 2010). Preliminary results in a cohort of 12 aMC individuals using the what-was-where task reported here also revealed significantly larger number of swap errors compared to controls at long delays, but normal performance in remembering the identity of the fractals (Liang, Pertzov, Crutch, Fox, & Husain, 2013). Investigation of the sensitivity of this task to normal aging was an important goal of this study. The finding that binding errors are not significantly affected by age suggests that it might have potential for early screening and monitoring response to treatment in AD, provided careful analysis is performed on the cause of swap errors.

Object-location misbinding rates, when corrected for selecting the identity of an object correctly by chance, might be suitable for distinguishing early deficits due to AD from those of normal aging. It is possible that they might be a more sensitive measure than color-shape binding because it has been shown that the hippocampus is already affected in preclinical stages of AD (De Leon et al., 1993; Fox et al., 1996; Jack et al., 1998). The hippocampus has been shown to be necessary for long term binding (Brasted, Bussey, Murray, & Wise, 2003; Kirwan & Stark, 2004; Yonelinas, Hopfinger, Buonocore, Kroll, & Baynes, 2001) as well as short term object-location binding (Olson, Page, Moore, Chatterjee, & Verfaellie, 2006; Piekema, Kessels, Mars, Petersson, & Fernández, 2006) such as used in this study (Pertzov et al., 2013). Therefore, it might be speculated that object-location binding impairment in visual STM can be specifically related to pathological changes underlying AD and might be a sensitive measure in preclinical stages of the disease. This possibility has to be further explored.

## Conclusions

Our main conclusions are that elderly people are worse than young people on both the recognition of object identity and recall of object location. Localization deficit was apparent even when only one item had to be remembered and worsened disproportionately when additional items were introduced. The age-related impairments did not escalate with longer retention intervals, implicating encoding or retrieval rather than maintenance processes. We also found an increased rate of swap errors in older people but this could be explained by their impaired memory of the objects’ identity rather than by impairment in object-location binding processes.

In future research, these findings should be compared with the patterns underlying STM decline in patients at early stages of AD. This might help to better characterize the differences between age-related and pathological cognitive decline.

## Supplementary Material

10.1037/a0038396.supp

## Figures and Tables

**Table 1 tbl1:** Summary Demographics of All Participants Binned Into Four Equal-Sized Groups According to Age

Age group		1	2	3	4
*N*		34	35	35	35
Age	Mean (*SD*)	25.2 (4.0)	35.2 (2.6)	49.2 (5.9)	67.5 (5.4)
	Range	19–31	31–40	40–60	60–83
Sex	M/F	18/16	18/17	13/22	13/22
Education (years)	Mean (*SD*)	15.6 (3.2)	16.9 (2.5)	15.0 (2.9)	15.3 (3.8)
MMSE	Mean (*SD*)	29.6 (0.5)	29.4 (1.0)	29.7 (0.6)	29.5 (0.8)
Digit span	Forwards	9.6	9.7	9.6	10.5
	Backwards	7.5	7.9	7.6	7.0
*Note.* M = male; F = female; MMSE = Mini Mental Status Examination.
