# Supplementary material for: Effects of Healthy Ageing on Precision and Binding of Object Location in Visual Short Term Memory
Source: Psychol Aging. 2014 Dec 22;30(1):26–35. doi: 10.1037/a0038396 (PMC4360752; doi:10.1037/a0038396)
Supplement: Supplementary file 1 [file z2m004142844so1[1].docx]

**Supplemental Materials**

**Effects of Healthy Ageing on Precision and Binding of Object Location in Visual Short Term Memory**

**by Y. Pertzov et al., 2014, *Psychology and Aging***

**http://dx.doi.org/10.1037/a0038396**

**Practice\learning effects**

One question of interest for us concerns the ability of participants to learn to bind objects to location with time. Does binding get better with practice? We were interested to study how age influences this ability to improve and if there is a difference between single-feature memory and binding processes in this respect.

Participants performed two blocks of 50 trials each. For the purpose described above we introduced the factor of block to the mixed-design ANOVA that now includes the factor of age-group (1 to 4) as between subjects factor and experimental block (1^st^ and 2^nd^), delay-duration (1s and 4s), and number-of-items (fractals; 1 and 3) as within subject measures. Next we report the results of this ANOVA that involve block effects or interactions. The rest of the effects and interactions are reported in the main text of the manuscript.

**Object identification**

Object identification performance (Figure S1) was computed by dividing the number of trials in which the target was identified correctly by the total number of trials. The mixed-design ANOVA showed an overall effect of learning between blocks [F(1,135)=7.4, p<0.01, $\eta_{p}^{2}$ = 0.05] reflecting an improvement in object recognition during the course of the task.

Additionally there was a significant interaction between block and number of items [F(1,135)=6.9, p<0.05, $\eta_{p}^{2}$ = 0.05] suggesting that the learning effect between the blocks is stronger when more items have to be remembered.


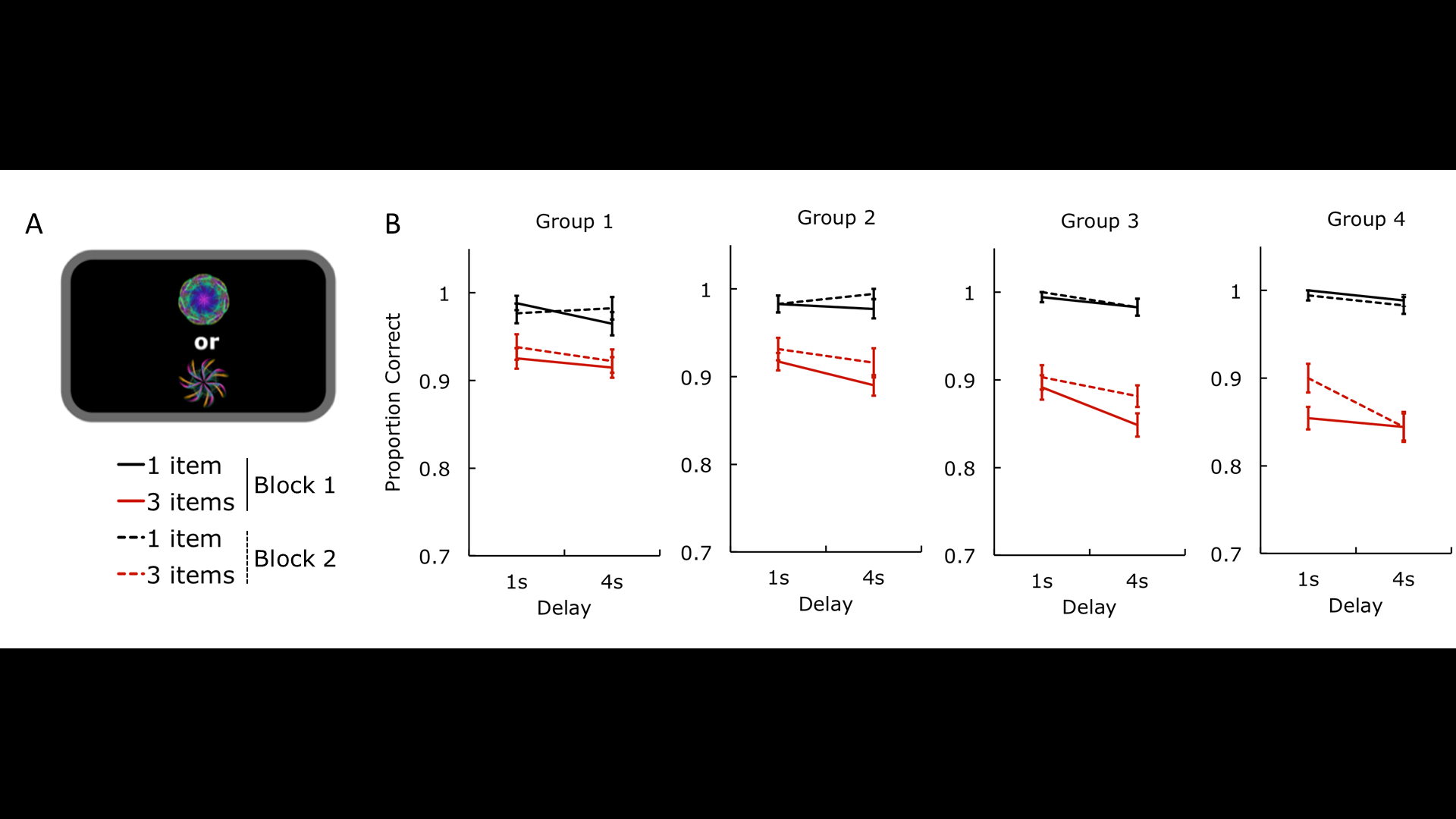


***Figure S1*. Identification accuracy**. **(a)** Schematic representation of the two-alternative forced-choice identification task. **(b)** Proportion of trials where the target was identified correctly shown for each of the 4 different age groups. The x-axis represents the delay duration. Straight lines represent block 1 and dashed lines block 2. Black lines denote 1 item conditions; red ones indicate 3 item conditions.

**Localization performance**

**Absolute localization error**

Absolute localization error (Figure S2) was measured as the difference between the reported location of a fractal and its true location in the memory array. Error decreased in the second block [F(1,135)=68.4, p<0.001, $\eta_{p}^{2}$ = 0.34].

There were also a significant block x delay [F(1,135)=5.4, p<0.05, $\eta_{p}^{2}$ = 0.04], block x items [F(1,135)=60.5, p<0.001, $\eta_{p}^{2}$ = 0.31] and block x delay x items interactions [F(1,135)=11.6, p<0.01, $\eta_{p}^{2}$ = 0.08], suggesting that the observed learning effects were more pronounced for longer retention intervals and/or more items to-be-remembered.


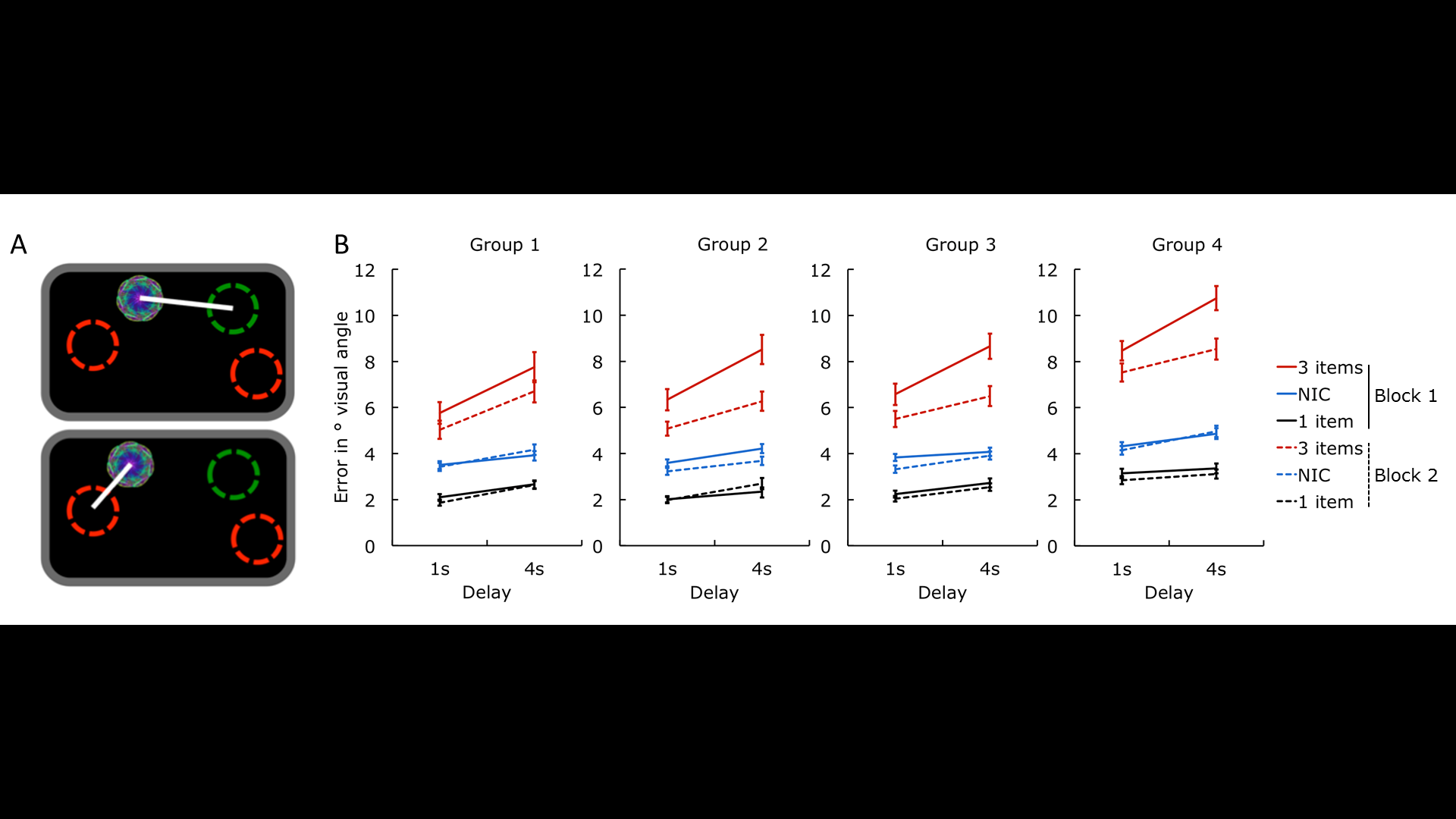


***Figure S2*. Localization performance. (a)** Schematic representations of the different measures (upper figure: error in a 3-items trial; below: NIC to the closest object. Green circles represent the target’s original location and red circles the non-target locations). **(b)** Localization error for the 4 different age groups: The x-axis represents the maintenance delay. Straight lines represent block 1 and dashed lines block 2. Red are 3 items conditions; Black are 1 item conditions; Blue are nearest item control (NIC) measures.

**Localization error controlling for swap errors**

Next we wanted to know how precisely people remember locations *regardless of the identity* of items. For this, we computed a measure of precision taking into account the fact that observers might sometimes identify the correct fractal but relocate it to a position of one of the other (non-probed) items in memory, or a non-target. We classify these mistakes as “swap errors”. Such swap errors would elicit big error values for absolute localization performance (= distance to target location) despite the fact that location of the chosen non-target position might be remembered with high precision. From this perspective, the actual localization error reflects not a lack of precision for the target location, but a decision to report the location of a non-target item. Therefore we computed the distance between the chosen location and the location of the *closest fractal* in the original memory array, regardless of whether or not it was the target (Figure S2). We term this measure ‘*nearest item control*’ (NIC).

Similar to raw localization performance, the NIC analysis revealed significant effects of block [F(1,135)=7.3, p<0.01, $\eta_{p}^{2}$ = 0.05], furthermore there was a significant block x delay interaction [F(1,135)=4.9, p<0.05, $\eta_{p}^{2}$ = 0.04], suggesting that learning effects were stronger in trials where the maintenance interval was prolonged. Additionally there was a significant three-way interaction between block x items x age-group [F(3,135)=6.3, p<0.001, $\eta_{p}^{2}$ = 0.12] suggesting that the learning effect is modulated by age when more items have to be remembered.

**Swap errors**

We attempted to explicitly quantify participants’ swap error rates by counting the trials where the target was correctly identified but subsequently localized within a radius of 4.5**°** visual angle of one of the non-target positions. Recall that each item in the memory array was separated from another one by a minimum of 9**°** degrees, so 4.5**°** provides a conservative window to classify swap errors.

We found a significant effect of block [F(1,135)=25.2, p<0.001, $\eta_{p}^{2}$ = 0.16], demonstrating a learning effect with fewer swap errors in the second block. In addition there was a significant block x delay interaction [F(1,135)=6.0, p<0.05, $\eta_{p}^{2}$ = 0.04] indicating that increased swap errors with longer delays were more pronounced in the first block.


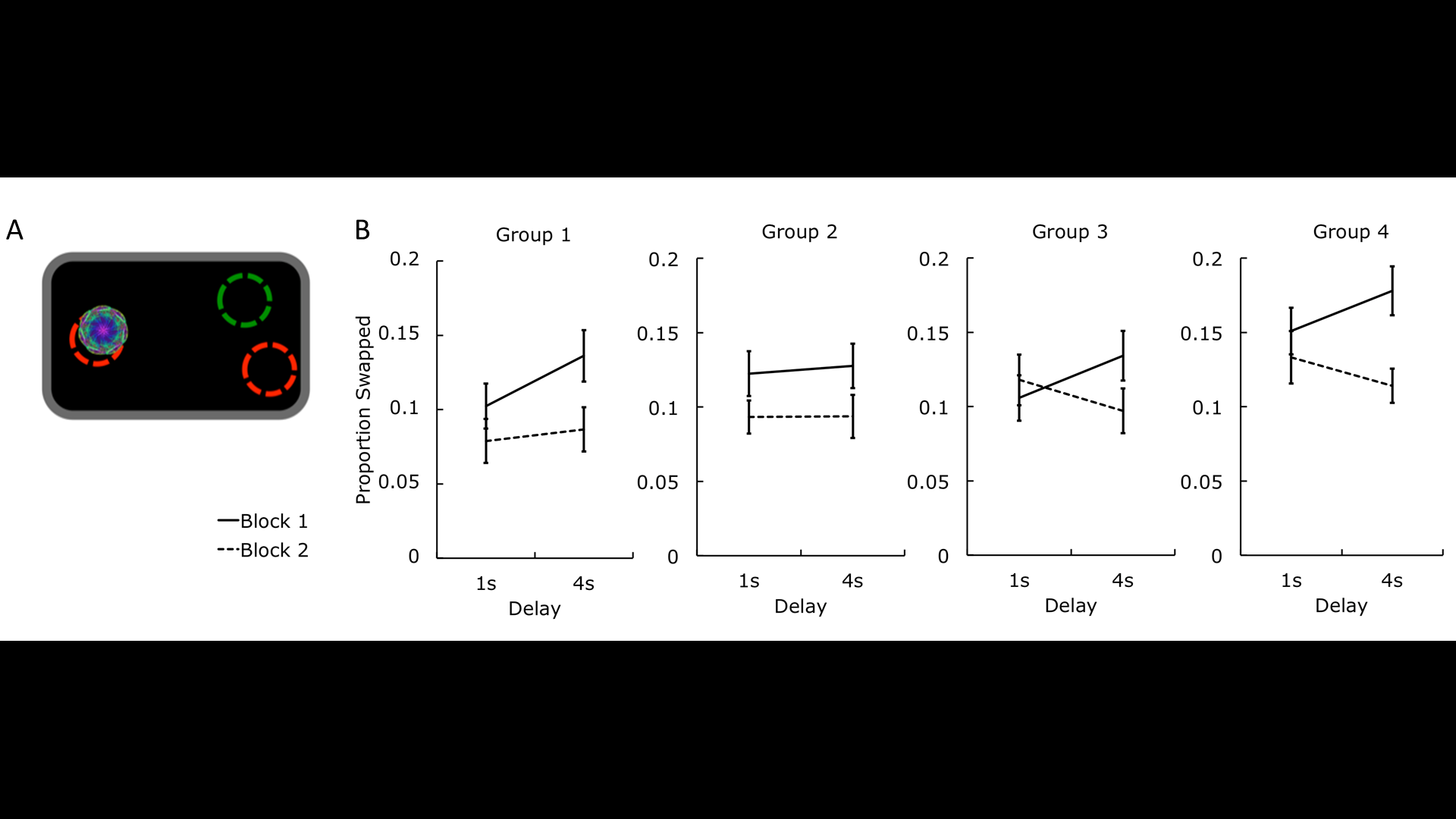


***Figure S3*. Swap errors for different age groups in first and second blocks of experiment.** (a) Schematic representation of a swap error (green circles represent the target’s original location and red circles the non-target locations). (b) Proportion of trials where the target has been located to a non-target location for the 4 different age groups. The x-axis represents the maintenance delay. Straight lines represent block 1 and dashed lines block 2.

**Discussion**

Although learning does improve object identification, localization, and the number of swap errors, no age-related differences in learning effects were found on either measure. Learning effects were generally stronger when the memory load was increased. However, this was true for all age group.
